# Caged-Sphere Optofluidic Sensors: Whispering Gallery Resonators in Wicking Microfluidics

**DOI:** 10.3390/s22114135

**Published:** 2022-05-29

**Authors:** Nicolas Riesen, Zane Q. Peterkovic, Bin Guan, Alexandre François, David G. Lancaster, Craig Priest

**Affiliations:** 1 Future Industries Institute, STEM, University of South Australia, Mawson Lakes, SA 5095, Australia; petzq001@mymail.unisa.edu.au (Z.Q.P.); bin.guan@unisa.edu.au (B.G.); francois.alexandre.au@gmail.com (A.F.); david.lancaster@unisa.edu.au (D.G.L.); craig.priest@unisa.edu.au (C.P.); 2 ARC Research Hub for Integrated Devices for End-User Analysis at Low-Levels (IDEAL), Future Industries Institute, STEM, University of South Australia, Mawson Lakes, SA 5095, Australia; 3 Institute for Photonics and Advanced Sensing, University of Adelaide, Adelaide, SA 5005, Australia

**Keywords:** resonators, integrated optics devices, biological sensing and sensors

## Abstract

The rapid development of optofluidic technologies in recent years has seen the need for sensing platforms with ease-of-use, simple sample manipulation, and high performance and sensitivity. Herein, an integrated optofluidic sensor consisting of a pillar array-based open microfluidic chip and caged dye-doped whispering gallery mode microspheres is demonstrated and shown to have potential for simple real-time monitoring of liquids. The open microfluidic chip allows for the wicking of a thin film of liquid across an open surface with subsequent evaporation-driven flow enabling continuous passive flow for sampling. The active dye-doped whispering gallery mode microspheres placed between pillars, avoid the use of cumbersome fibre tapers to couple light to the resonators as is required for passive microspheres. The performance of this integrated sensor is demonstrated using glucose solutions (0.05–0.3 g/mL) and the sensor response is shown to be dynamic and reversible. The sensor achieves a refractive index sensitivity of ~40 nm/RIU, with Q-factors of ~5 × 10^3^ indicating a detection limit of ~3 × 10^−3^ RIU (~20 mg/mL glucose). Further enhancement of the detection limit is expected by increasing the microsphere Q-factor using high-index materials for the resonators, or alternatively, inducing lasing. The integrated sensors are expected to have significant potential for a host of downstream applications, particularly relating to point-of-care diagnostics.

## 1. Introduction

Optofluidic sensor platforms are being intensely studied for their unique combination of optical precision and sensitivity and lab-on-a-chip ease-of-use and efficiency [1,2,3]. Many recently reported optofluidic devices exploit the straight-forward integration of optical components (light emitting diodes, lenses, optical filters) and fluidics (microfluidic channels, capillaries, droplets) [4,5,6]. However, some of the most promising optical and fluidic phenomena are not easily integrated, due to incompatibility of physical or chemical constraints, and, when achieved, are anchored to the laboratory by the necessary infrastructure [7]. This paper presents a new concept for an optofluidic sensor which does not require precision pumps and allows for free-space whispering gallery mode (WGM) sensing that is ultimately suitable for portable devices.

The underlying sensing technique for this novel platform is whispering gallery modes (WGMs) which occur in resonators that have a circular symmetry whereby light is trapped inside by total internal reflection [8,9,10,11,12]. The light circulates along the inner surface of the resonator, returning in phase after each round trip and hence satisfying the resonance conditions. In a WGM resonator, the spectral positions of the resonances depend not only on the resonator geometry, but also on the surrounding environment. WGMs are therefore ideally suited for refractive index (RI) sensing [8]. We note here that RI sensing is a particularly attractive sensing methodology as the signal scales with the analyte concentration rather than the volume, making it suitable for small volume applications. RI sensing is also label-free, relying on the intrinsic properties of the target species and thus requiring fewer reagents and sensing steps.

To exploit the use of WGMs, two approaches generally exist. In the most common approach, evanescent field coupling between a phase-matched optical fibre taper (or prism) and the resonator is used [13]. While this approach has allowed for single-molecule detection and the realisation of enormous Q-factors [11,14,15,16], the use of thin optical fibre tapers is particularly problematic, whereas the use of prisms is difficult for smaller (higher-sensitivity) resonators [8]. The alternative approach is to use resonators with a gain medium incorporated. When the so-called active resonator is illuminated by a remote light source, the gain medium emits light [8] and, due to the Purcell effect, there is an increase in the emission rate of the gain medium at the resonance wavelengths. This means that the fluorescence spectrum appears with modulated peaks corresponding to the microcavity resonances [17]. The wavelength shifts of these resonances can then be used to infer changes in the liquid.

This indirect WGM coupling scheme is particularly attractive for in situ or point-of-care applications since it is robust and practical, requiring only free-space pump light illumination of the resonators. The WGM Q-factors are, however, typically 3 to 4 orders of magnitude lower than fibre taper or prism-coupled passive resonators [17], implying that these active resonators tend to have inferior detection limits. These detection limits are, however, typically sufficient for the majority of practical applications (i.e., ng/mL to mg/mL [8]) that do not, for instance, require single-molecule detection. With research into active resonators still in its infancy, there remains significant opportunities for closing the performance gap. One example of a method for enhancing the Q-factors is by inducing lasing, which effectively increases the energy storage capacity of the microcavity [18]. Since the practical advantages are unmatched, active resonators open the door to new applications not previously possible [8].

Several different platforms have been reported in the literature for integrating whispering gallery mode resonators on a sensing chip. Often, WGM resonators are immobilised onto a chip or they form part of the microfluidic channel, and a driven force usually a pump is required to deliver the analyte into the area with the WGM resonators for detection. For example, a WGM microgoblet array was sealed inside a polymer microfluidic channel for RI sensing, while a peristaltic pump was used to inject analyte solutions into this microfluidic system [19]. Other examples include integrated microspheres for flow cytometry [20], and fluorescence-labelled microspheres for multiplexed biosensing [21].

The microfluidic component of the optofluidic sensor reported here is based on an open micropillar array system where liquid can spontaneously fill into the void space between pillars via a capillary action called wicking, and form a well-defined film [22,23,24]. The thickness of the liquid film is precisely determined by the height of the pillars (in µm), and the volume required to fill the microfluidic chip is of the order of several µL [22]. The pillar array, similar to other open microfluidic systems, has advantages over traditional enclosed systems in terms of simple sample loading, rinsing and cleaning, and controllable evaporation can be used to drive a continuous flow between the pillars [25]. It was reported by Orlowska et al. that by prefilling the pillar array with a known liquid, a predictable flow behaviour driven by evaporation of the liquid could be achieved, including for a biological fluid such as saliva or sweat [25].

In this paper, we report on the integration of active microsphere WGM resonators into this open microfluidic chip platform, demonstrating a practical and high-sensitivity refractive index sensing-based optofluidic system. By caging the microsphere in the forest of pillars, the position of the sphere is maintained without being influenced by the flow of liquid or being subject to random motion. Meanwhile, the evaporation-driven lateral flow enables continuous sampling and monitoring.

## 2. Experimental Setup

### 2.1. Active WGM Microspheres

Polystyrene microspheres (Polysciences Inc., Warrington, PA, USA) with nominal diameter of 15.0 µm, standard deviation of 0.6 µm, and refractive index of 1.591 were used. The fluorescent dye incorporated in the microspheres was Nile Red (Sigma Aldrich, St. Louis, MO, USA, λ_abs_ ~ 532 nm, λ_em_ ~ 590 nm) using a two-phase technique [18]. In this technique, the Nile Red is dissolved in xylene up to the solubility limit. This solution is then poured into an aqueous solution of microspheres and stirred with a magnetic stirrer until the xylene has evaporated. Since xylene and water are immiscible and the fluorescent dye is hydrophobic, the fluorescent dye is transferred into the microspheres on contact when the xylene evaporates. After the microspheres are doped, the solution is heated at 95 °C for 1 h to remove the solvent. The microspheres are then washed by centrifugation, and the supernatant removed [18].

### 2.2. Open Microfluidic Chip Fabrication

The microfluidic chips were fabricated in fused silica using standard photolithography and plasma etching techniques (see Supplementary Materials, Method S1: Fabrication of microfluidic chips). The microfluidic chip designed and used within the paper, unless mentioned otherwise, has a square lattice of pillars with a diameter of 10 µm, height of 20 µm and lattice spacing of 20 µm. The analysis area (i.e., the pillar array) of the chip (7 mm × 12 mm) is approximately 6 mm × 8 mm.

**Figure 1:**
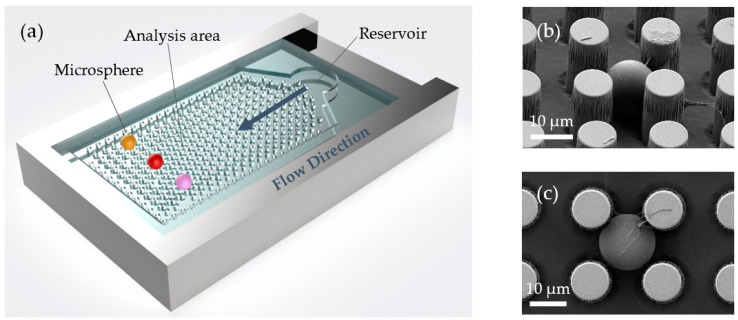
(**a**) Schematic of the open microfluidic chip (not to scale) with caged whispering gallery microspheres incorporated. (**b**,**c**) SEM micrographs of the polystyrene microspheres caged within the open microfluidic chip.

The integration of microspheres into the chip was realised via the following steps. A 3 µL droplet of diluted microsphere solution was first loaded into the middle of the array using a pipette and allowed to diffuse through the pillars. A brief torrent from an ultra-high purity nitrogen flow from a handheld gun was used to dry the chip. The microspheres (typically 10 or more within the pillar array) remained firmly lodged within the microfluidic chip with only point contacts with the pillars that were found to have negligible impact on the microspheres’ Q-factors. The microfluidic chips were optimised such that the pitch of the pillars is ideal for capturing the microspheres. This removes the challenge of tracking spheres which otherwise would be perturbed by liquid flowing over them. A schematic of the open microfluidic chip (not to scale) is shown in Figure 1a with SEM micrographs (Zeiss Merlin Field-Emission Gun SEM) of microspheres caged between pillars shown in (b) and (c).

### 2.3. Interrogation Setup

A 532 nm CW laser source (JDS Uniphase, Milpitas, CA, USA, Model 21006959) was coupled into the back port of an inverted microscope (IX 71, Olympus, Tokyo, Japan) equipped with a 532 nm dichroic mirror effectively using the microscope in a confocal arrangement. The free-space excitation of the microspheres in the microfluidic chip was achieved through the inverted microscope with a 20× objective, using the 532 nm laser as the pump source (see Figure S1: Experimental setup photo). The focal length of the objective is ~3 mm. The emission was collected back through the same objective and coupled into a 200 µm patch fibre. In this proof-of-concept demonstration, we used a non-planar configuration with perpendicular orientation of the incoming and outgoing light. In principle it would be possible to integrate optical waveguides and LED sources into the plane of the chip to realise a planar device.

**Figure 2:**
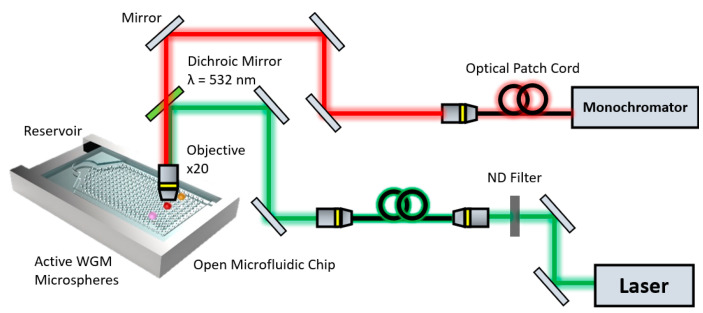
Experimental setup using inverted microscope for interrogation of whispering gallery microspheres integrated into the open microfluidic chip.

The WGM modulated fluorescence spectrum from a given microsphere was then spectrally resolved by a monochromator (iHR550, Horiba, Kyoto, Japan) equipped with a 1200 lines/mm, 500 nm blaze grating and a thermoelectrically cooled CCD (Synapse 2048 pixels, Horiba, Kyoto, Japan). The experimental setup is shown in Figure 2. A series of glucose solutions ranging in concentration from 0.05 g/mL to 0.3 g/mL were then prepared (uncertainty of <5%). Glucose was chosen since the refractive index is well known and linear with respect to the concentration, making it ideal for characterising the resonance-shift as a function of refractive index [26].

## 3. Results and Discussion

### 3.1. WGM-Based Sensing of Single Glucose Concentrations

The sensing principle of this optofluidic sensor was demonstrated with a series of glucose solutions. In the first series of tests, a new microfluidic chip and microsphere was used for each individual glucose concentration measurement.

**Figure 3:**
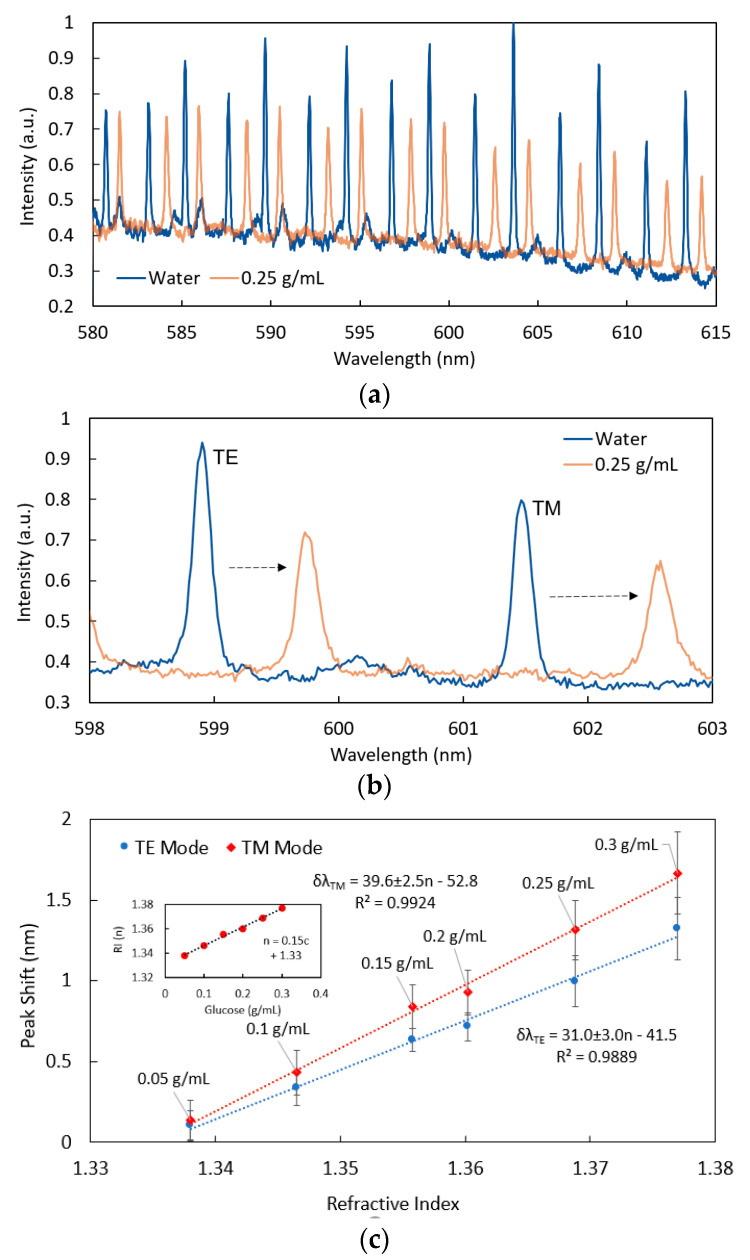
(**a**) Typical normalised WGM-modulated fluorescence spectrum (blue: pure water, orange: 0.25 g/mL glucose in water) of 15 µm microspheres (FSR ~ 4.8 nm) lodged in an open microfluidic chip. (**b**) Wavelength shift of isolated TE and TM resonances from grey-dashed box in (**a**). (**c**) Wavelength shift of TE and TM modes within the microfluidic chip as a function of refractive index. The glucose concentration was converted to refractive index (RI) at 633 nm using [26], with the calibration shown in the inset. In these measurements, a different sphere and chip was used for each measurement. The water in the chip for the baseline measurement was also evaporated before the glucose solution was added.

To optically characterise this integrated WGM microsphere-based sensor, purified water (3 µL) was first loaded into the reservoir of the chip and allowed to wick across the surface of the analysis area (6 mm × 8 mm). A microsphere with suitably high-quality WGMs was then located and the spectrum was recorded under 532 nm laser excitation of ~200 µW. The water in the microfluidic chip was then left to evaporate, before a given concentration solution of glucose (3 µL) was loaded into the reservoir. With the microsphere immersed in the solution, the spectrum was recorded for the given glucose concentration and the shift of the resonance peaks towards longer wavelengths (i.e., red shift) compared to pure water was determined (Figure 3a,b). This process was then repeated for each of the remaining glucose concentrations, using new microfluidic chips and new microspheres for each measurement, avoiding the requirement for rinsing. A total of three measurements were taken and averaged for each glucose concentration.

Readers should note that within the timeframe of the measurements undertaken and at ~200 µW, no significant photobleaching of the Nile Red dye in the microspheres was observed. It is also worth noting that predominantly the first-order Transverse Electric (TE) and Transverse Magnetic (TM) polarisation whispering gallery modes are observed for the resonator in aqueous solution although higher-order modes with lower intensity and lower Q-factor (λ/λ_FWHM_) appear to be present as seen in the spectrum of Figure 3a. The resonance wavelength positions were determined using Lorentz fits on the resonance peaks. Typical Q-factors of around 5 × 10^3^ were calculated for the first-order TE and TM modes (λ_FWHM_ ~ 120 pm at 600 nm), which is comparable with previous findings [8].

The resonance wavelength shift at ~ 600 nm for the TE and TM modes relative to pure water for each of the individual glucose concentrations (average of three measurements, error given as S.D.) was plotted against the refractive index of the glucose solution, as shown in Figure 3c. The response is clearly linear in the range considered as is expected since the relative change in refractive index is approximately equal to the relative change in resonance wavelength, i.e., δn/n = δλ/λ [8]. The TM polarisation has higher sensitivity, as might be expected due to the associated weaker confinement of light within the resonator and hence stronger evanescent field [18]. The refractive index sensitivities of the TE and TM modes are 31 ± 3 nm/RIU and 40 ± 3 nm/RIU, respectively, and this is comparable with previously reported values [8,18]. The red-shift in the resonances occurs due to a decrease in the confinement of light in the microspheres as the index contrast decreases. This in turn results in an increase in the optical pathlength (i.e., longer wavelength) necessary to satisfy resonance.The detection limit (LoD) was calculated based on the more sensitive TM modes which had a sensitivity of 39.6 ± 2.5 nm/RIU. Wavelength shifts in the resonance peaks equivalent to one full-width half-maximum (FWHM) can readily be determined using Lorentz fits, and this was used as the basis to determine the LoD. The FWHM from the definition of Q-factor is given by:

(1) $$\lambda_{FWHM}=\frac{\lambda }{Q}$$

The LoD can then be given as:

(2) $$\mathrm{LoD}=\frac{1}{{\delta \lambda }_{TM}}\lambda_{FWHM\;}=\frac{\lambda }{{\delta \lambda }_{TM}Q}$$

when using the lower limit of the refractive index sensitivity (i.e., 37.1 nm/RIU) and given the Q-factor of the WGM modes was typically ~5 × 10^3^ at 600 nm, this leads to a LoD of 3 × 10^−3^ RIU (~20 mg/mL glucose). Improvement of the Q-factor using lasing [18,27] or by using higher quality microspheres could see detection limits further enhanced to around 10^−4^ RIU, an upper limit imposed by the 4 pm resolution limit when using the 2400 lines/mm grating of the monochromator. The variability observed in the refractive index sensitivity can be accounted for by the use of different-size microspheres (15.0 µm ± 0.6 µm) for different glucose measurements. The wavelength Free Spectral Range (∆λ_FSR_) was used to confirm that the diameters of the microspheres were within the standard deviation prescribed by the manufacturer using:

(3) $$\Delta \lambda_{FSR}\approx \frac{\lambda^{2}}{2\pi nR}$$

where λ = 600 nm is the fluorescence wavelength, n = 1.59 is the refractive index of the microsphere at λ and R is the microsphere radius. Along with the red-shift of the resonances with increasing glucose concentration, we also observed a reduction in Q-factor. This was a result of a decrease in WGM confinement and increase in evanescent field when the refractive index contrast between the microsphere and its surrounding decreases. The trend was approximately a reduction in Q-factor of ~200–300 per 0.01 RIU change.

### 3.2. Continuous Sampling and Monitoring with Evaporation-Driven Flow

In the second series of tests, a microfluidic chip preloaded with water was used for the glucose measurements in an attempt to further understand the behaviour of the microfluidic system. In these tests, the liquid in the analysis area was not allowed to evaporate before adding the next glucose concentration to the reservoir. This served to simulate repeated use of the microfluidic system and assisted in understanding the platform’s behaviour as relevant to continuous real-time RI measurements. A single microfluidic chip and microsphere was thus used for all glucose concentrations.

As previously reported, evaporation-driven flow that is insensitive to interfacial tension and viscosity of a sample fluid can be realised in micropillar arrays [25]. If the pillar array is long enough and the reservoir is inexhausted, a continuous flow can be achieved. To realise simple sample manipulation and create a continuous flow in the open microfluidic chip, a longer microfluidic chip with a 15 mm wide × 70 mm long array of pillars was used for these glucose measurements. The longer chip allows for the previous liquid sample to be pushed further away from the WGM microsphere when a new liquid is added to the reservoir, avoiding any potential mixing during the measurement. As the water evaporates, the glucose concentrates towards the end of the pillar cuvette, allowing the device to operate over long periods before saturation of solute occurs.

In these tests, a microsphere-laced chip was loaded with 10 µL of purified water through its reservoir, and a single high-quality WGM microsphere was located approximately 30 mm down the chip. This was followed by applying a 7 µL droplet of the 0.05 g/mL glucose solution to the reservoir with a pipette, when the preloaded water in the reservoir had evaporated (approximately 1–2 min) but the analysis area was still wet. The spectrum of the microsphere was then measured within a few seconds. The fluid in the chip reservoir driven by evaporation was allowed to completely flow into the pillar array over approximately 1–2 min and then, while the analysis area was still wet, 7 µL of the 0.10 g/mL glucose solution was applied.

**Figure 4:**
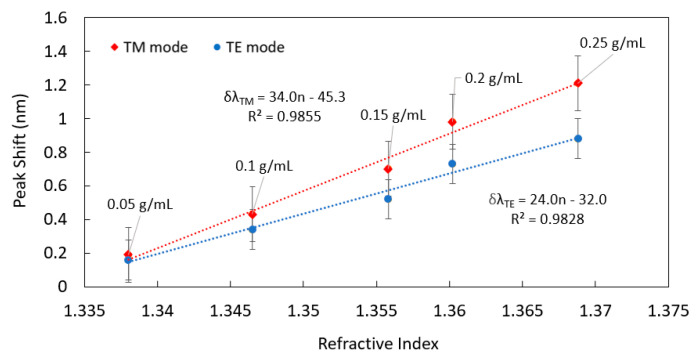
Wavelength shift of TE and TM WGMs within a longer microfluidic chip, as a function of glucose concentration. In this result progressively higher concentrations of glucose are added to the chip reservoir rather than allowing the liquid to first evaporate. In this case, a single chip and microsphere was used for all measurements.

Immediately, the spectrum of the microsphere was recorded again. This process was then repeated for the higher concentrations, ensuring at each step that the chip reservoir had expired, to avoid mixing or dilution of the applied solutions. This approach is different to the previous methodology in that it uses the same sphere and chip for all measurements and may be suitable for continuous measurements. It also does not require the previous sample to be rinsed or washed out of the chip. Both advantages come from having the longer chip, in which the previous liquid is pushed away towards the end of the chip where it concentrates. The resonance wavelength shift for the TE and TM modes at around ~600 nm as a function of the glucose concentration is shown in Figure 4. The response observed is again linear with higher sensitivity for the TM modes as expected. The gradients of the linear fits differ from the previous set of results from Figure 3c by up to 25% because the location of the sphere with respect to the reservoir differed. Since there is an approximately exponential increase in glucose concentration along the chip length [25], this means that the position of the sphere along the chip is important for calibration purposes and it influences the sensitivity of the chip. The longer pillar array has the advantage of the previous glucose solution being transported far from the sphere location, which ensures that the next measurement is not affected by the previous. This allows the caged-sphere platform to be used repeatedly or continuously.

**Figure 5:**
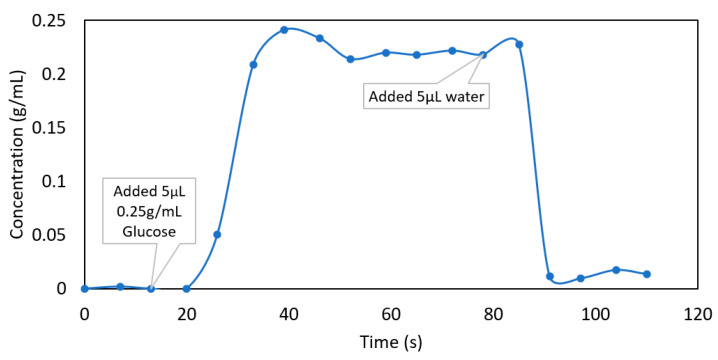
In-chip dynamics monitored for the microfluidic chip pre-loaded with water, as a 5 µL droplet of 0.25 g/mL glucose was added to the reservoir. The result showcases the potential of the microfluidic caged-sphere platform for practical real-time monitoring of liquids.

The in-chip fluid dynamics are also of particular interest and are analysed in Figure 5. This figure shows a times series of the in-chip glucose concentration determined from the wavelength shift of in this case a TM mode resonance at ~600 nm, as a 5 µL 0.25 g/mL concentration glucose droplet was added to the water-loaded chip. In this time series, measurements were taken at 6.5 s intervals over 115 s, with the result clearly showing a “front” of high-concentration glucose flowing across the WGM microsphere. Importantly, the shift in resonance peak is also reversible when pure water is again added into the reservoir. In Figure 5, the response time is ~15 s; however, the sensor’s response time can be shortened by reducing the distance between the sphere and the reservoir or enhancing the evaporation rate. Figure 5 therefore clearly demonstrates the power of the WGM microspheres, not only for characterising the behaviour and fluid dynamics of the open microfluidic chip, but also for potential real-time or continuous analysis of sample concentrations. Importantly, this platform does not require mechanical pumps and could be implemented without bulk components.

### 3.3. Cycling and Reversibility Tests

There is the possibility of slowly flowing liquid into the chip reservoir with auxiliary microfluidic channels and undertaking real-time continuous monitoring of the concentration of a given liquid. This is possible because the WGM resonance shift is reversible, as shown in the previous section. Here, we further demonstrate the potential of the technique by undertaking cycling tests where the concentration of glucose is increased and decreased multiple times in the chip with specific concentrations fed to the reservoir via a pipette.

**Figure 6:**
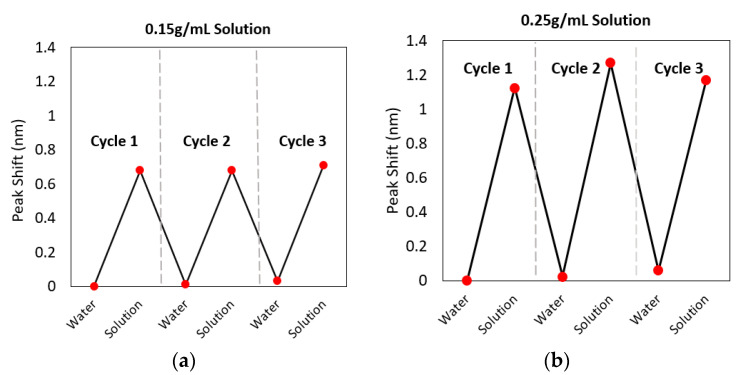
Cycling tests between water and (**a**) 0.15 g/mL or (**b**) 0.25 g/mL glucose solutions in the chip. The TM resonance wavelength (at ~600 nm) response is seen to be reversible, opening up the opportunity for continuous monitoring of the refractive index of liquids using this platform.

First, the spectrum of a microsphere located in a water-loaded microfluidic chip (the longer chip with 15 mm × 70 mm analysis area) was recorded. A 7 µL droplet of the glucose solution was then added to the reservoir in the same manner as described in the previous section and the spectrum was measured. This was followed by applying a 10 µL droplet of water to the reservoir after 1 min and then re-measuring the spectrum. The resonance shifts of three repeats of the water-glucose cycles are shown in Figure 6 for the case of glucose concentrations of (a) 0.15 g/mL and (b) 0.25 g/mL. These results clearly show that the resonance wavelength shift is reversible and repeatable. This occurs because the liquid added into the reservoir pushes the previous liquid or glucose solution towards the end of the microfluidic chip which is seen as a deposited high-concentration band. The behaviour of the open microfluidic system that enables continuous sampling can also be visualised using colour dye instead of the transparent glucose solution.

**Figure 7:**
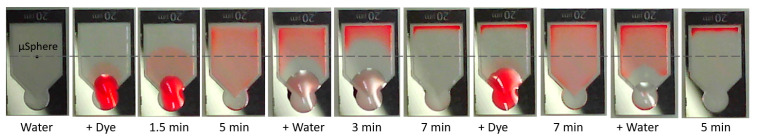
Visual demonstration of the behaviour of the microfluidic system (the 7 mm × 12 mm chip) using Ponceau-4R colour dye.

Figure 7 demonstrates the wicking and washing cycles and, as is expected, the dye is pushed towards the end of the chip, where it concentrates when the droplet of water is added. This permits continuous sampling, with the new liquid sample flushing out the previous. These results further demonstrate that real-time monitoring of liquids could be possible using this caged-sphere platform. The elimination of the requirement for mechanical pumping, and the longer timescales for evaporative flow (up to 10 s seconds), could allow for extended monitoring of environmental or biological samples in a compact device. We note, however, that some prior knowledge of the sample may be necessary given that multicomponent solvent mixtures may complicate the interpretation of measurements. A fixed solvent matrix is, however, not an unrealistic assumption in many applications. The microfluidic approach can be used for sensing independent of temperature and humidity, as shown elsewhere [25]. It is envisioned that bioconjugation of the microspheres could allow for specific binding of a variety of target analytes including proteins, bacteria and DNA [8]. The use of multiple beads located on the microfluidic chip surface each with unique functionalisation could also allow for future multiplexed biosensing demonstrations.

## 4. Conclusions

In conclusion, we have demonstrated a practical integrated optofluidic chip platform that could be suitable for WGM biosensing. The platform uses pump-free evaporation-driven microfluidic chips to deliver liquid to active microsphere resonator sensors. The use of active resonators having a gain-medium incorporated avoids the use of cumbersome optical fibre tapers and allows for free-space excitation and illumination of the whispering gallery modes. The integrated platform was characterised using different glucose concentrations exhibiting a refractive index sensitivity of ~30–40 nm/RIU and a detection limit of 3 × 10^−3^ RIU. The in-chip behaviour of the sensing platform was also investigated demonstrating that real-time monitoring of liquid flow would be possible. This is because the refractive index of a liquid can be measured continuously with this platform (increase and decrease in the refractive index). With conjugation of the microspheres for specific binding and potential integration of waveguides or light sources into the chips, it is expected that this sensing platform could lead to a host of practical and high-sensitivity integrated biosensing applications.

## Data Availability

The data presented in this study is available on request from the corresponding author.

## Supplementary Materials

The following are available online at https://www.mdpi.com/article/10.3390/s22114135/s1, Figure S1: Photo of experimental setup, Method S1: Fabrication of microfluidic chips.
