# Supplementary material for: Caged-Sphere Optofluidic Sensors: Whispering Gallery Resonators in Wicking Microfluidics
Source: Sensors (Basel). 2022 May 29;22(11):4135. doi: 10.3390/s22114135 (PMC9185560; doi:10.3390/s22114135)
Supplement: Supplementary file 1 [file sensors-22-04135-s001.zip › sensors-1740524-supplementary/SupplementaryInformation/Method S1.pdf]

sensors

Supplementary Information

Method S1: Fabrication of microfluidic chips

A 6-inch diameter fused silica wafer (Shin-Etsu Quartz Products Co., Ltd.) was first deposited with 100 nm of Cr via sputtering (HHV TF500) which serves to stop glass reflection of UV light during the photolithography process. It was then spin coated with SU8-10 photoresist at 2500 rpm for 30 s. After pre-exposure baking at 65 °C for 2 min and 95°C for 5 min, the SU8 photoresist layer on the silica wafer was exposed to UV (360 nm, EVG 620) at a dose of 150 mJ/cm^2 through a chromium-glass photomask of circular patterns in hard contact mode.

Post-exposure baking was conducted at 65 °C for 1 min and 95 °C for 2 min and the SU8 patterns on the wafer were developed in propylene glycol monomethyl ether acetate for 2 min, prior to hard baking at 130 °C for 10 min. Plasma etching (ULVAC, NLD570) based on Ar and C_3F_8 gases was carried out on the SU8-patterned wafer to sequentially remove the exposed Cr and etch the silica to a required depth. The remaining Cr on the silica wafer was removed by 10% (w/v) ceric ammonium nitrate solution, rendering totally transparent open microfluidic chips.
