# Supplementary figures and images for: Caged-Sphere Optofluidic Sensors: Whispering Gallery Resonators in Wicking Microfluidics
Source: Sensors (Basel). 2022 May 29;22(11):4135. doi: 10.3390/s22114135 (PMC9185560; doi:10.3390/s22114135)

## Supplementary Information

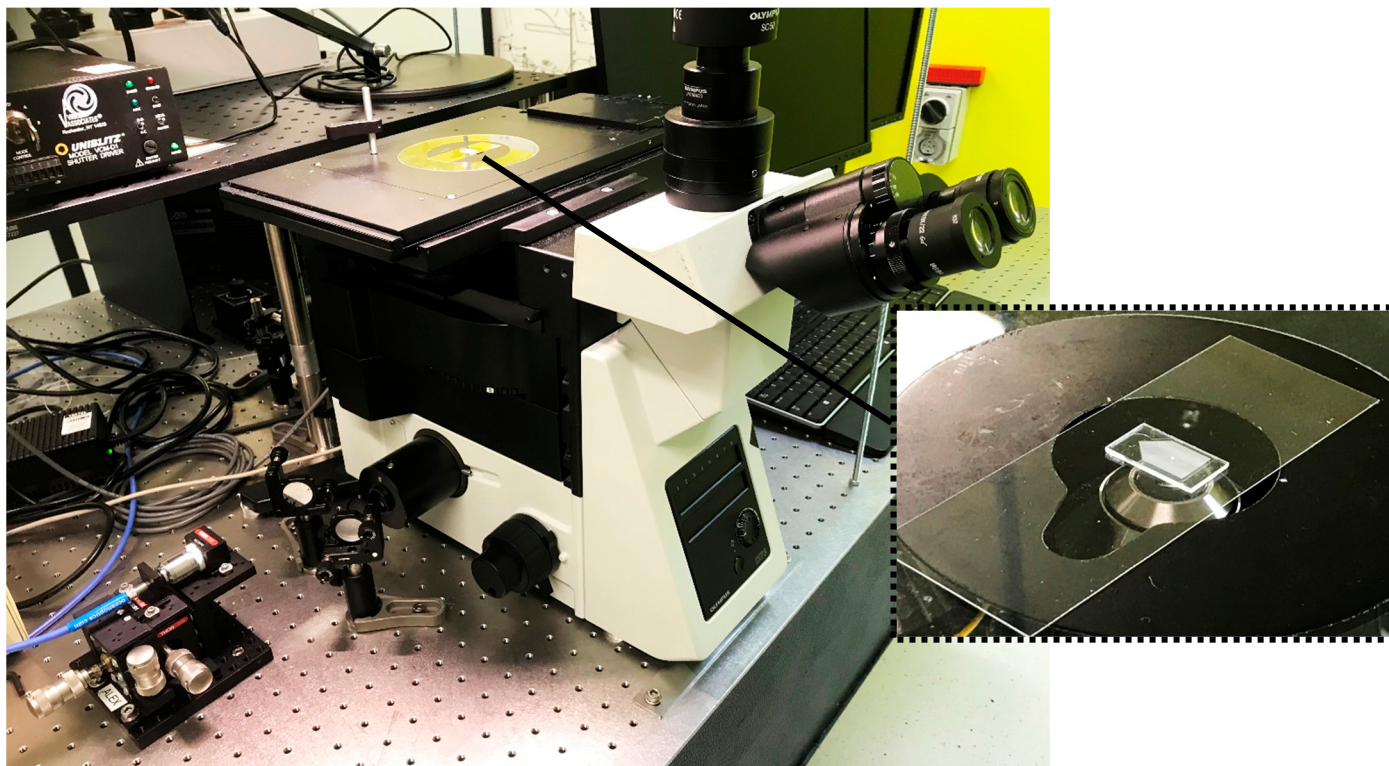

**Figure S1.** Photo of experimental setup.

Supplement: Supplementary file 1 [file sensors-22-04135-s001.zip › sensors-1740524-supplementary/SupplementaryInformation/Figure S1.pdf]
